# A Glimpse of Molecular Biomarkers in Huntington’s Disease

**DOI:** 10.3390/ijms23105411

**Published:** 2022-05-12

**Authors:** Silvia Martí-Martínez, Luis M. Valor

**Affiliations:** 1 Servicio de Neurología, Hospital General Universitario Dr. Balmis, Instituto de Investigación Sanitaria y Biomédica de Alicante (ISABIAL), 03010 Alicante, Spain; smmcyv@coma.es; 2 Laboratorio de Apoyo a la Investigación, Hospital General Universitario Dr. Balmis, Instituto de Investigación Sanitaria y Biomédica de Alicante (ISABIAL), 03010 Alicante, Spain

**Keywords:** polyglutamine, huntingtin, CSF, plasma, saliva, NF-L, PDE10A, cytokines, transcriptomics, miRNA, oxidative, somatic expansion

## Abstract

Huntington’s disease (HD) is a devastating neurodegenerative disorder that is caused by an abnormal expansion of CAG repeats in the Huntingtin (*HTT*) gene. Although the main symptomatology is explained by alterations at the level of the central nervous system, predominantly affecting the basal ganglia, a peripheral component of the disease is being increasingly acknowledged. Therefore, the manifestation of the disease is complex and variable among CAG expansion carriers, introducing uncertainty in the appearance of specific signs, age of onset and severity of disease. The monogenic nature of the disorder allows a precise diagnosis, but the use of biomarkers with prognostic value is still needed to achieve clinical management of the patients in an individual manner. In addition, we need tools to evaluate the patient’s response to potential therapeutic approaches. In this review, we provide a succinct summary of the most interesting molecular biomarkers that have been assessed in patients, mostly obtained from body fluids such as cerebrospinal fluid, peripheral blood and saliva.

## 1. The Need for Biomarkers in Huntington’s Disease

With an estimated prevalence of 1–15 cases per 100,000 inhabitants [1], Huntington’s disease (HD) (OMIM #143100) is a monogenic neurodegenerative disorder that similarly affects both sexes and is caused by an abnormal expansion of CAG repeats in the first exon of the Huntingtin (*HTT*) gene. Although more information is needed regarding the physiological function of the HTT protein, it acts as a scaffolding protein to facilitate multiple protein interactions in different processes, such as vesicle and endosomal trafficking, cell division and survival, and the regulation of autophagy and transcription [2]. Aberrant CAG expansion causes the production of an excessively long polyglutamine (polyQ) fragment that exerts its toxic effects within the cell by abnormally interacting with key components of diverse cellular processes, inhibiting their activity or sequestrating them into mutant huntingtin (mHTT) aggregates, together with the consequences of the loss of a functional allele [3]. More recently, other toxic HTT-derived molecules have been described; expanded CAG transcripts and derived small CAG (sCAG) RNA induce gene silencing, DNA damage and apoptosis [4,5,6], whereas cleavage of mHTT also produces non-polyQ fragments that provoke endoplasmic reticulum vacuolation and stress [7]. More controversial is whether the production of polyA, polyS, polyL and polyC peptides from repeat associated non-AUG (RAN) translation processes has deleterious effects [8,9].

Despite discovering the cause of HD almost thirty years ago [10], there is still no cure, and current therapeutic regimes are focused on treating specific symptoms, such as chorea (with the use of tetrabenazine or derivatives) and psychiatric disorders (with the use of antidepressants and anxiolytics) [11]. Classical HD is manifested by a complex symptomatology that can be classified into the following main domains [12]:

1. Motor impairment, including extrapyramidal motor impairments such as chorea (sudden involuntary dyskinetic movements) which is the main characteristic feature of the disease that appears in >90% of patients in the first ~10 years of the disease, but it is gradually replaced by bradykinesia, rigidity and dystonia. Other symptoms are oculomotor impairments (e.g., slow saccadic and ‘bird head’ movements), disability of voluntary motor function (clumsiness), hyperreactive reflexes, abnormal gait, speech deficits leading to mutism (with preserved language), dysphagia and swallowing problems;
2. Cognitive impairment, including executive function impairment at the level of planning and judgement (disorganization, impulsivity, lack of flexibility), difficulties in simultaneous tasks, attention deficits, general psychomotor retardation, manipulative disability of the egocentric space, inefficient memory but with the capability to create new memories and to retain information, deficits in learning mainly of motor skills, among others;
3. Psychiatric symptoms, including depression, inactivity, apathy, insomnia, mania, delusions of grandeur, anxiety and irritability, aggressive behavior, disinhibition, schizophrenia-like signs (e.g., auditive hallucinations, emotional blunting), etc.

In general, the temporal course of the disease usually starts with mood changes and mild cognitive problems, followed by more apparent motor abnormalities (chorea) and worsening of psychiatric and cognitive disorders. Later, weight loss, sleep disturbance, incontinence, bradykinesia and rigidity are the most relevant symptoms until death 10–20 years from the onset of disease, mainly caused by pneumonia and cardiovascular incidents, but suicide is also frequent. Furthermore, other clinical variants can show different manifestations of the disease, as in the case of juvenile HD (<20 years, 5–10% of cases), a rigid variant with Parkinsonian-like motor impairment, along with seizure attacks and emotional problems and Westphal HD (20–30 years), which is also a mainly rigid variant, and with milder and slower progressive late onset HD (>50 years).

The *HTT* gene encodes a ubiquitous protein that is expressed in nearly all tissues, except in mesenchymal cell types, showing high expression levels in the central nervous system (CNS) (see also https://gtexportal.org/home/gene/HTT#geneExpression (accessed on 19 April 2022)) [13]. Irrespective of mHTT expression, most of the symptoms can be explained by the dysfunction and degeneration of the basal ganglia and the corticostriatal circuitry [14], in which the GABAergic medium spiny neurons (MSN) of the corpus striatum are especially sensitive to mHTT expression. Although the reasons for this sensitivity are still unclear, some explanations have been proposed; for example, these neurons do not endogenously express brain neurotrophic factor (BDNF) and their survival is highly dependent on the BDNF supply from the cortex, which is disrupted in the disease [15], and the somatic instability of the CAG expanded allele (which is tightly associated with the age of symptomatology onset [16,17]) is more pronounced in the striatum than in other tissues [18]. These and other factors can combine with the intrinsic striatal susceptibility to mitochondrial oxidative stress [19]. Nonetheless, the effect on other brain areas (e.g., the cortex, hippocampus, hypothalamus and cerebellum) also impacts the quality of life of patients [20,21,22]. In addition, there is increasing evidence of a peripheral component of the disease that can be linked to the direct effects of mHTT, although the real impact on patients remains to be fully explored [23,24,25,26]: weight loss; muscle atrophy; reduction of the bone mineral density; cardiac and respiratory dysfunction; changes in the gastrointestinal microbiota; endocrine dysfunction; platelet abnormalities; elevated levels of peripheral proinflammatory cytokines, etc. It is well established that the number of CAG repeats inversely correlates with the age of onset of the disease in the clinical population but the relationship between the number of CAG repeats and survival has been less explored. Despite first observations indicating that the rate of progression to death was largely independent of the number of CAG repeats [27], new studies are challenging this view. In the most recent report based on the REGISTRY clinical data, the authors realized the need to control for age of disease onset to enable an appropriate analysis, finding that longer CAG expansions were predictive of shorter survival for a fixed age of onset [28]. In any case, the relationship between the CAG repeats and disease outcomes shows a high dispersion within patients and is insufficient to explain the large variability in the pleiotropic manifestation of the symptoms [29,30]. This fact, together with a long pre-manifested period that is still poorly characterized [31] with documented cases in which brain atrophy and structural changes take place prior to overt symptomatology [32,33], justifies the search for biomarkers with a prognostic value in HD, to improve our understanding of the pathological mechanisms of disease and to predict the progression and age of onset of symptoms [34]. Therefore, such biomarkers will facilitate decision-making in the clinical management of individual patients and will serve to evaluate the response to potential novel therapeutic approaches.

A biomarker is defined as any biological feature or trait that can be measured to evaluate undergoing physiological or pathological processes or to evaluate responses to therapeutic interventions. An optimal biomarker provides measurements that should be objective, reproducible, sensitive, specific and obtained with minimal invasiveness [35]. To identify novel biomarkers and enhance our knowledge about pathological mechanisms, peripheral fluids and tissues are examined as biomaterial sources, taking special consideration to cause minimal discomfort to those patients with low wellbeing during the sample extraction. In the case of HD, peripheral blood has been the preferred source of biomarker discovery, followed by cerebrospinal fluid (CSF) [36]. Brain constituents are released into the CSF and, in most cases, subsequently into the bloodstream as a consequence of axonal damage, neuronal death or secretion increase. These molecules can be quantified using immunodetection assays, such as enzyme-linked immunosorbent assay (ELISA), electrochemiluminescence (ECL) or single molecule array (SIMOA), with the latter generally being the most sensitive [37]. Although CSF is the most accessible way to analyze the CNS milieu, lumbar puncture has an inherent risk, increases discomfort in HD patients and evokes reluctance towards the procedure; in addition, CSF biomarkers also show substantial inconsistencies across studies, as do other body fluids [38]. In this review, we will provide a brief overview of the most interesting molecular biomarkers in HD.

## 2. Huntingtin

HTT and mHTT can constitute biomarkers by themselves. Despite its predominant intracellular localization, HTT can be physiologically secreted into the CSF, as well as mHTT in pathological conditions, due to the removal of brain extracellular solutes by the glymphatic system (an aquaporing-4 water channel-based waste drainage system), as documented in animal models [39]. Thanks to this system, it is feasible to quantify soluble mHTT in CSF, of which the concentration is higher in symptomatic than pre-symptomatic individuals and in later stages of the disease, and correlates positively with the motor score (namely, the Unified HD Rating Scale or UHDRS) and cognitive performance [40]. Despite these observations, mHTT monitoring does not improve the prognostic performance of other neurodegenerative markers that are easy to detect [41] (see below), but can still be useful to assess mHTT-lowering therapeutics [42].

In contrast, the detection of mHTT in plasma is challenging. Nonetheless, mHTT can be monitored in the cell fraction of blood, more precisely in peripheral monocytes and T and B cells, which can contain significant differences between manifest and premanifest expansion carriers [43]. Among leukocytes, the mHTT levels of monocytes showed a better correlation with caudate atrophy and disease burden [43].

## 3. Biomarkers of Neuronal Injury

### 3.1. NF-L

Neurofilaments are type IV intermediate filaments and are integral constituents of neurons that are mainly located in the myelinated axons of the CNS and the peripheral nervous system (PNS). They enable neuronal cytoskeleton plasticity, maintain neuronal architecture, ensure axonal integrity and improve the speed of neural transmission. Neurofilaments are classified according to their molecular weight as heavy (NF-H), intermediate (NF-M) and the most soluble light chain (NF-L). NF-L is the most promising wet biomarker in HD for both disease onset and progression and can be determined in CSF and plasma/serum [37,44,45,46]. Its increase in both CSF and blood has been largely observed in various conditions: Alzheimer’s disease (AD); Parkinson’s disease (PD); typical Parkinsonism; frontotemporal dementia; multiple sclerosis and amyotrophic lateral sclerosis (ALS), Creutzfeldt–Jakob disease, etc. [34,44]; therefore, NF-L is a general marker of neurodegeneration, brain trauma and neuroinflammation. In HD, NF-L levels are correlated with brain atrophy and both motor and cognitive decline [34]. Although NF-L levels are easier to be detected in CSF than in plasma, collection of plasma is less invasive for donors once it has been demonstrated that there is a good correlation between CSF and plasma levels for this protein [45,47]. Compared to mHTT levels, NF-L discriminates better between pre-symptomatic and symptomatic stages and shows good correlations with brain volumetrics and clinical features of HD [41,48]. Moreover, plasma NF-L predicts the age of disease onset in the following three years in pre-symptomatic patients [47].

### 3.2. Tau

Tau is primarily expressed in the axon of neurons and is involved in microtubule assembly and stabilization, neurite outgrowth and synaptic plasticity [49]. Phosphorylation and dephosphorylation are key mechanisms to regulate tau activities; they are disrupted in AD and tauopathies [50,51], leading to hyperphosphorylation of tau, destabilization of microtubules and formation of fibrils and aggregates that contribute to cellular dysfunction and death [51,52,53,54]. From the first reports indicating alterations of tau in the brains of HD patients [55,56], there is appealing evidence of tau hyperphosphorylation, increased total levels, mis-splicing affecting the 4R-Tau/3R-Tau ratio, protein misfolding and aggregation, subcellular redistribution, neurofibrillary tangles and neuropil threads in the brains of HD patients [57,58] that appears to correlate with late cognitive deficits and dementia in HD patients [56,59], suggesting that HD may have a tauopathy component [60] that deserves further exploration. As a biomarker, an increase in total tau in CSF has been found in HD patients [61] and can be regarded as a nonspecific marker of neuronal damage, although its prognostic value seems to be weaker than NF-L [62].

### 3.3. TDP-43

The transactive response (TAR) DNA binding protein of 43 kDa (TDP-43) is the main pathological protein in ALS and the most common subtype of frontotemporal dementia (FTD), concurring with ubiquitylated inclusions [63]. TDP-43 is a ubiquitous RNA/DNA-binding protein that belongs to the heterogeneous nuclear ribonucleoprotein (hnRNP) family and regulates RNA metabolism, mRNA transport, microRNA maturation, autophagy and stress granule formation, among others [64]. The presence of TDP-43 in CSF is explained by synapse degeneration and loss [65]. Noticeably, a small proportion of patients (<0.2%) affected by either FTD or ALS conditions also showed CAG expansions in the *HTT* gene in the pathological range in the absence of HD-associated striatal atrophy, suggesting a potential etiopathological relationship between HTT mutation and other degenerative disorders [66]. As a biomarker, TDP-43 levels in the plasma of HD patients seem to correlate with the presence and severity of apathy and with cortical thinning of the frontal and anterior-temporal regions [67].

## 4. Other Neural-Derived Biomarkers

### 4.1. PDE10A

Cyclic nucleotide phosphodiesterases (PDEs) control the intracellular concentrations of the second messengers cAMP and cGMP by hydrolyzing them into the inactive forms 5′cAMP and 5′cGMP; therefore they are key regulators of cyclic nucleotide signaling [68] and have been implicated in diverse neurodegenerative conditions, including HD [69]. PDE10A is predominantly expressed in the brain (with the highest expression in the striatum) and becomes profoundly deregulated in the basal ganglia areas of HD mouse models and postmortem brains of patients, which may explain the deficiencies in cAMP-dependent signaling and associated CREB transcriptional activity [70,71,72,73]. Of note, the critical role of PDE10A in the control of striatal-dependent movement is demonstrated by mutations in the coding sequence of the *PDE10A* gene that cause infantile-onset limb and orofacial dyskinesia (IOLOD, OMIM #616921), in which there is a reduction of the protein in the striatum [74], and autosomal dominant striatal degeneration-2 (ADSD2, OMIM # 616922), in which the activity of PDE10A is affected, more precisely the binding to cAMP to stimulate the PDE10A catalytic domain [75]. Both conditions are characterized by hyperkinetic, dyskinetic and choreic movements that are accompanied by striatal atrophy in the case of ADSD2.

The use of specific radioligands ([18F]MNI-659, [11C]IMA107) allows for the visual monitoring of PDE10A levels in the brains of HD patients by positron emission tomography (PET). The reported studies demonstrated alterations in the levels of this phosphodiesterase that can occur in pre-symptomatic stages prior to overt brain atrophy and correlates with several parameters of disease progression with improved accuracy compared to volumetric measurements [76,77,78,79]. PDE10A levels are not only reduced in striatal structures but are also altered in other brain areas (augmented in the motor thalamic nuclei [79], decreased in the insular cortex and occipital fusiform gyrus [80]), which may be associated with specific phenomena of the disease.

### 4.2. BDNF

BDNF has emerged as a key factor in neuropathology, mostly in psychiatric disorders and neurodegenerative processes [81,82], and can be measured in the plasma and serum of patients affected by these conditions [83]. In HD, the gene expression of cortical Bdnf is reduced, explaining the reduction of striatal protein in mouse models and human postmortem brains [15,84]. Some results indicated that serum BDNF was reduced in manifested HD patients compared to healthy controls [85], consistent with a reduction in *BDNF* mRNA [86]; however, neither its transcript nor protein showed differences in other studies between controls, pre-symptomatic and symptomatic individuals [82,87,88], as several factors can influence BDNF concentration (e.g., sex, age, inter assay variations, instability of BDNF after plasma/serum collection and preparation) [83]. In addition, BDNF production from platelets can act as a confounding factor as it follows different dynamics [89]. This lack of differences was also extended to CSF [88]. In contrast, the BDNF promoter IV methylation in blood cells was increased in HD patients, showing an inverse correlation with anxiety and depression scores [87]. Whether this alteration may be useful as a marker of psychiatric symptoms in HD deserves further validation.

### 4.3. Neuropeptide Y

Striatal projection MSNs account for ~95% of striatal neurons, and their progressive dysfunction is responsible for some of the symptoms found in HD patients, affecting the dopaminergic D2-type indirect pathway of movement in early stages of the disease. GABAergic neurons expressing neuropeptide Y (NPY) are a fraction of the remaining ~5% of interneurons that are relevant in the regulation and synchronization of MSN activity. With a molecular size of 36 amino acids, NPY is one of the most abundant neuropeptides of the brain, and it participates in the regulation of food intake, energy homeostasis, circadian rhythm, cognition and stress, and has neuroprotective effects [90,91]. In HD, immunoreactivity of NPY is augmented not only in the basal ganglia but also in other brain areas such as the cortex and the subventricular zone [92,93,94], indicating an increase in the number of NPY-expressing cells, likely in an attempt to compensate for the malfunction of the affected neuronal cell subtypes. It has been recently proposed that this increase is reflected in the levels of NPY in the CSF of HD patients compared to control donors, although without discriminating disease stages [95].

## 5. Markers of Oxidative Stress

Oxidative stress and mitochondrial dysfunction are key in the progression of late stages of HD [34]. Several molecules have been analyzed and reviewed elsewhere that are pending further validation [45]. One interesting marker of the reduced antioxidant capacity observed in the disease is the low concentration of uric acid, a product of purine metabolism with antioxidant properties in the CNS and PNS [96,97], in the plasma and saliva in HD patients compared to control donors, that may reflect the reduced levels observed in postmortem brains [98].

The kynurenine pathway is involved in the oxidative metabolism of tryptophan in microglia that generates metabolites such as 3-hydroxykinurine (3-HK) and its downstream product quinolinic acid (QUIN), which exert potent neurotoxic effects by inducing excitoxicity through NMDA receptors and increasing the production of reactive oxygen species (ROS) that induces lipid peroxidation, among other actions [99]. In contrast, another metabolite of the same pathway, kynurenic acid (KYNA), has neuroprotective effects, and manipulation of the kynurenine pathway to produce KYNA instead of 3-HK and QUIN has been postulated as a potential therapeutic approach [100]. However, the presence of these three metabolites in CSF has apparently no prognostic value, as no differences were observed between controls, premanifest and manifest patients [101].

## 6. Transcriptional Profiling of Peripheral Blood

As previously mentioned, brain malfunction and neurodegeneration in HD are evoked by the disruption of multiple cellular processes, including transcription [102,103,104]. Transcriptional dysregulation is an early event, as demonstrated in cellular preparations in which gene expression changes occur prior to cell loss, mHTT aggregation or mitochondrial dysfunction, and in animal models with minimal phenotypical and morphological manifestations of the disease, but showing a prominent disrupted transcriptome that worsens during progression (reviewed in [103]). Gene expression changes in the brains of mouse models and patients are mostly conserved [70,71,105]. Nonetheless, transcriptional dysregulation associated with HD is not restricted to the CNS but can also be found in nonneuronal tissues, such as blood [106], muscle [107,108] and diverse peripheral tissues [72] in HD animal models. Therefore, it is reasonable to apply transcriptomics’ approaches to the analysis of blood samples and skin fibroblasts from patients, which initially supported distinctive profiles between pre-symptomatic and symptomatic stages and identified potential biomarkers for measuring both disease progression and response to treatment (based on histone deacetylase inhibition) [109,110,111,112]. Using microarray and DeepSAGE technologies, several biomarkers have been proposed: *AIM2*; *ANXA1*; *ANXA3*; *AQP9*; *ARFGEF2*; *BCL2L1*; *CAPZA1*; *CYSTM1*; *DAB2*; *DUSP1*; *EGR1*; *GOLGA8G*; *H2AFY*; *HIF1A*; *HLA-DQA1*; *IER3*; *IL1B*; *IL8*; *LPAR6*; *LTBR*; *MARCHF7*; *MDX1*; *MLH3*; *NEAT1*; *NFKBIA*; *P2RX1*; *PCNP*; *PPIF*; *PPP1R15A*; *PROK2*; *ROCK1*; SAP30; *SF3B1*; *SP3*; *SUZ12*; *TAF7*; *TNF*; *TNFRSF17*; *YPEL5* or *ZNF238* [109,110,111,112,113,114,115,116,117]. However, even using the most updated transcriptomics technology based on next-generation sequencing (NGS) in the form of RNA-seq [118], there is a lack of replication across studies using peripheral blood as a biomaterial source. In addition, no clear association was observed with disease progression within 2 years of a longitudinal study [116]. In an effort to reconcile these studies, Andrade-Navarro, Priller and colleagues compared their own RNA-seq results with previous reports [119], suggesting that a HD blood signature may exist after all, as some overlaps were detected (e.g., *ANXA3*, *CYSTM1*, *IL1B* and *PROK2*). This lack of validation can be accounted for by confounding demographic and individual factors in natural human populations, which are critical in studies using small cohorts, low standardization of screening platforms and analytical tools, sample collection and storage and blood heterogeneity. Moreover, despite the expression of mHTT in blood cells [43], its impact on gene expression is not as profound as that in neurons. Thus, the reported differential expression in peripheral blood between HD manifest patients and healthy controls was limited to a few genes compared to brain structures (none or very few after FDR correction [116,118,120]). In the study by Hensman-Moss et al. [118], the dysfunction of pathways rather than genes seems to be shared between brain and blood, an observation that is difficult to translate into the clinics. Nonetheless, there is evidence in favor of a relevant role of white blood cells in trinucleotide expansion disorders, such as the increase of proinflammatory factors in blood with prognostic value (see below), the increased apoptosis induced by mitochondrial stress of lymphoblasts derived from HD patients [121] and the hyper-reactivity of myeloid cells of HD patients and animal models [122,123].

MicroRNAs (miRNAs) are of special interest as biomarkers because they can be obtained not only from whole blood as cellular transcripts but also from the plasma and serum fractions [124], as they are strikingly stable as constituents of extracellular vesicles and lipoproteins and RNA-binding protein complexes [125,126]. In the case of HD, variations in circulating miR-10b-5p and miR-486-5p in plasma resemble those observed in brain miRNAs [127], certain miRNAs (miR-122-5p, miR-100-5p, miR-641 and miR-330-3p) can be associated with the functional capabilities of patients [128], and their levels can be modified after diet-based interventions (e.g., miR-338-3p, miR-128-3p, miR-23a-3p and miR-24-3p [129]). Other miRNAs have been found to be different between HD patients and healthy controls (e.g., miR-34b and miR-323b-3p [130,131]). However, these RNA species also suffer the same challenges of reproducibility and validation of mRNAs.

## 7. Immune Response and Inflammation Markers

Noticeably, searching for gene co-expression patterns rather than differential expression in the transcriptomes from the brain and blood of patients, immune response genes were found to be coregulated at the CNS and peripherally [118,132], pinpointing common mechanisms of neuroinflammation and peripheral inflammation. Using a proteomics approach, inflammatory-related proteins were found to be altered in the plasma of expansion carriers, such as complement components, α-2-macroglobulin and clusterin [133]. The same study also detected increased levels of proinflammatory IL-6 in the plasma of moderate HD patients compared to early, premanifest and control donors [133]. An independent study confirmed the increased levels of IL-6 in premanifest carriers, with an estimated average of 16 years before the age of motor onset [134]. Another documented early marker of inflammation was IL-8, whereas the anti-inflammatory IL-10 and IL-14 were increased at moderate stages, although significant correlations with UHDRS were found for IL-8 and TNF-α [134]. In contrast, immunoglobulin levels were unchanged. Chemokines are small chemoattractant cytokines that were explored in a posterior study, which reported elevated levels of the eosinophil-related eotaxin and eotaxin-3 and the monocyte/macrophage-related MIP-1β, MCP-1 and MCP-4 [135]. Of these, eotaxin, eotaxin-3 and MIP-1β were more associated with disease stage, whereas eotaxin and MCP-1 were better linked to motor scores and functional capacities [135]. Overall, these results support an activation of the innate immune cells without the involvement of the adaptive immune response system.

## 8. Saliva as an Alternative Source of Biomarkers in HD

Seeking novel sources of biomarkers, muscular biopsy has also been proposed, based on preclinical studies [107,136], but this procedure is highly invasive. Saliva has attracted recent attention due to studies reporting interesting biomarkers even in prodromal stages and for its noninvasiveness and low cost of sampling. Saliva is secreted in the mouth by the principal salivary glands to serve in nutrient digestion and buccal protection and consists of a complex heterogeneous fluid, mostly composed of water containing a mixture of electrolytes, mucopolysaccharides and glycoproteins, digestive enzymes (e.g., amylase and lipase), antimicrobial agents (e.g., secreted IgA and lysozyme) and cells, among other compounds. Apart from bacteria, cells are primarily epithelial squamous cells (keratinized, nonkeratinized and intermediate) and leukocytes (mature granulocytes followed by monocytes and T and B cells) [137,138]. The list of promising salivary biomarkers in neuropathology contains: Aβ42; Tau and lactoferrin in AD; α-synuclein and DJ-1 in PD; chromogranin A in ALS and a collection of miRNAs in autism spectrum disorder (ASD) [139]. Salivary levels of cortisol have been associated with depressive symptomatology and verbal memory deficits in early HD patients [140,141,142]. Saliva also contained increased levels of total HTT protein in HD patients compared to healthy controls, which significantly correlated with clinical outcomes measured using the UHDRS and total functional capacity (TFC) [143]. Moreover, decreased levels of salivary BDNF have been reported in pre-symptomatic and symptomatic patients compared to healthy donors [87]. Last, the salivary levels of uric acid correlated as well as plasma levels with the motor and functional capacity scores in HD patients [98]. Despite these observations requiring further validation in independent cohorts, saliva may constitute a promising noninvasive source of biomarkers that deserves further attention.

## 9. Genetic Modifiers as Biomarkers

As already discussed, the inverse correlation between the number of CAG repeats and the age of disease onset is far from being satisfactory. Noticeably, loss of the CAA triplet within the CAG tract of the *HTT* gene does mot modify polyQ length, but results in earlier disease onset, indicating that longer CAG alleles rather than longer polyQ length are better associated with disease onset [17], and are related to somatic instability [144]. Intense efforts have been dedicated to identify genetic modifiers that may somehow interact with the CAG expansion and modulate the age of onset and progression of the HD symptomatology. Several polymorphisms and genetic variations have been reported in the last few years, fueled by genome-wide sequencing approaches, that are located in genes such as *TCERG1* (*aka* CA150, *Transcription Elongation Regulator 1*) [145]; *UCHL1* (*Ubiquitin C-Terminal Hydrolase L1*) [146]; *GRIN2A* and *GRIN2B* (*Glutamate Ionotropic Receptor NMDA Type Subunit A/B*) [147]; *FAN1* (*FANCD2 and FANCI-Associated Nuclease 1*); *HERC2P10* (*Hect Domain And RLD 2 Pseudogene 10*); *RRM2B* (*Ribonucleotide Reductase Regulatory TP53 Inducible Subunit M2B*) and *UBR5* (*Ubiquitin Protein Ligase E3 Component N-Recognin 5*) [30]; *E2F2* [148]; *MSH3* (*MutS homolog 3*); *DHFR* (*Dihydrofolate Reductase*) and *MTRNR2L2* (*Mitochondrially Encoded16S RNA-Like 2*) [149]; *MLH1* (*MutL Homolog 1*) [150]; *PIAS1* (*Protein Inhibitor Of Activated STAT 1*) [151], *PMS1* and *PMS2* (*PMS1 Homologs 1/2*) [152], among others. Of these, the most promising candidates to serve as genetic modifiers (and as predictive biomarkers) are related to DNA repair activities, because they can regulate somatic expansion [18,153]. This is of interest, as somatic expansion increases over the life span and is not restricted to the brain as it is also observed in the blood [154]. This relation between polymorphisms associated with DNA repair loci and somatic expansion can be shared by other repeat expansion disorders [155].

## 10. Biomarkers in Clinical Trials

As most of the aforementioned biomarkers discussed in this review remain to be validated, their use in clinical trials has been quite limited (see [156] for an update on HD clinical trials). The most notable exception is the determination of mHTT levels in CSF as a secondary outcome in antisense oligonucleotide (ASO) strategies aimed at lowering mHTT, as in the case of the stereopure ASOs WVE-120101 and WVE-120102 (with the recent addition of WVE-003) that target SNPs associated with mutant *HTT* allele, and the nonallele-specific silencing IONIS-HTTRx/RG6042/tominersen compound. Biomarkers can also be therapeutic targets. This is the case with PDE10A, where further inhibition of PDE10A by the PF-02545920 drug has intriguingly beneficial effects in HD [157] and has been tested in patients (NCT02197130).

## 11. Conclusions

In recent years, an increasing number of biomarkers were proposed to predict the age of onset and severity of the disease in HD. Validation of these candidates has usually been hampered due to the low prevalence of the disorder, making the recruitment of relatively homogeneous cohorts difficult. Of these, the detection of NF-L in blood and CSF seems to be one of the most suitable approaches to monitor the health status of HD patients, although peripheral measurement of IL-6 appears to be a good candidate to consider. More tightly linked to HD-specific dysfunction, examination of PDE10A offers the possibility for non-invasive imaging, but requires extensive preparation of individuals. In addition, the identification of genetic modifiers has opened the door for the prediction of disease outcomes in subgroups of patients, providing at the same time important clues regarding HD pathogenesis that can be druggable. Improving our understanding of the ethiopathological mechanisms triggered by CAG expansion of the *HTT* locus will also lead to proposals of novel strategies in biomarker screenings.

## Data Availability

Not applicable.
